# Publisher Correction: Recycled iron fuels new production in the eastern equatorial Pacific Ocean

**DOI:** 10.1038/s41467-017-02138-3

**Published:** 2017-12-05

**Authors:** Patrick A. Rafter, Daniel M. Sigman, Katherine R. M. Mackey

**Affiliations:** 1 Department of Earth System Science, University of California, Irvine, CA 92697 USA; 2 Department of Geosciences, Princeton University, Princeton, NJ 08540 USA

*Nature Communications* **8**:1100 10.1038/s41467-017-01219-7; Article published online: 24 October 2017

The original version of this Article contained errors in Fig. 2b and Table 2. In Fig. 2b, the white circle labels were incorrectly positioned as they referred to scenarios that were used in an earlier version of the Article. In Table 2, the following three sentences were removed from the legend ‘The last two calculations are discussed in the “Methods”. The first assumes that all dissolved plus the ≈0.3 nmol kg^−1^ of particulate iron (measured in the eastern equatorial Pacific^30^) is bioavailable. The last calculation assumes EUC dissolved iron concentrations from 140° W′. These errors have now been corrected in both the PDF and HTML versions of the Article.

