# Supplementary material for: Recycled iron fuels new production in the eastern equatorial Pacific Ocean
Source: Nat Commun. 2017 Oct 24;8:1100. doi: 10.1038/s41467-017-01219-7 (PMC5653654; doi:10.1038/s41467-017-01219-7)
Supplement: Supplementary file 1 — Supplementary Information [file 41467_2017_1219_MOESM1_ESM.pdf]

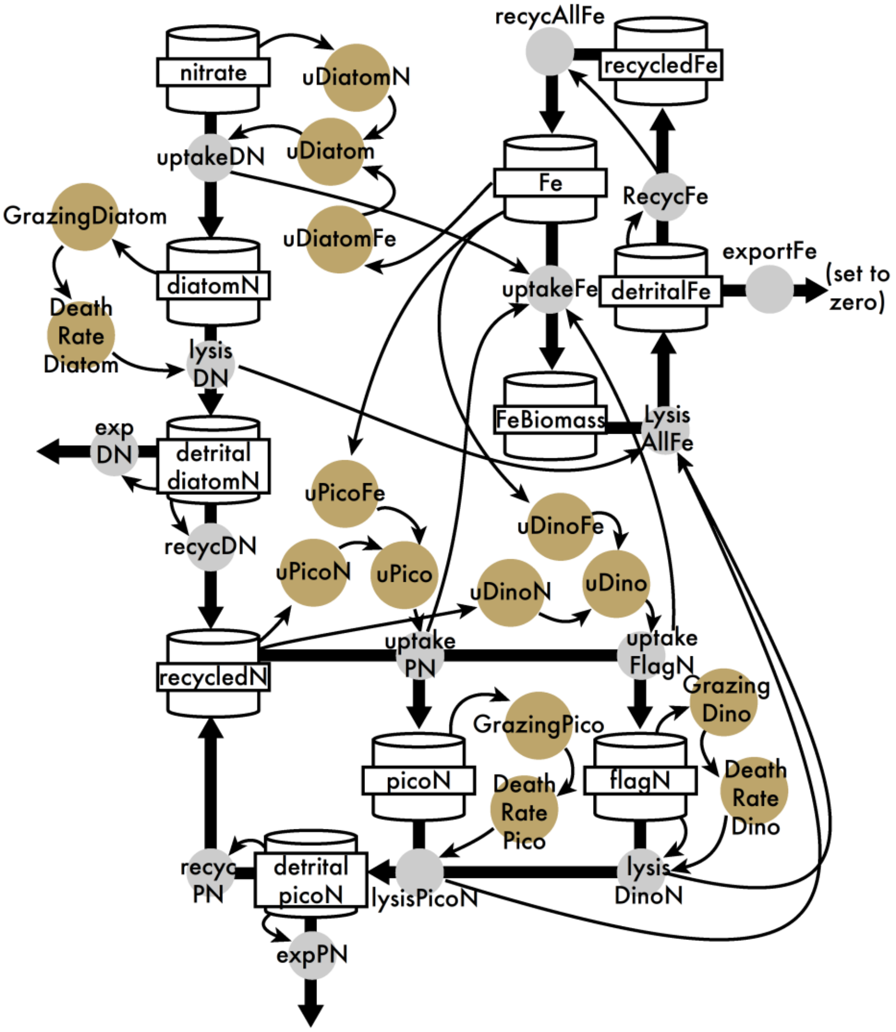

Supplementary Figure 1. Schematic for the eastern equatorial Pacific model of internal nutrient cycling. Cylinders (black and white titles) are reservoirs of nutrients, biomass, and detritus. Wide arrows illustrate flows between reservoirs and narrow arrows indicate connections between reactions (see equations in Supplementary Data). Gold spheres represent scalar values that drive flow rates (calculations in silver spheres) between reservoirs. Calculations beginning with "u" involve growth rate associated with each group's N and Fe uptake. Flow rates beginning with "uptake" involve nutrient uptake; "lysis" involve the combined grazing and mortality of phytoplankton; "recyc" involves recycling from the detrital pool; and "exp" involves export from a detrital pool. "Pico" refers to picoplankton and "Flag" refers to photosynthetic flagellates. See Methods and Supplementary Data for more details.

Supplementary Table 1. Values and references for parameters used in the internal nutrient cycling model. Parameters taken from Community Earth System Model-Biogeochemical Elemental Cycling Model settings [1] are marked CESM, but converted to a nitrogen budget. Rates from the CESM were halved to account for no diurnal cycle in our model. Settings for photosynthetic flagellates were assumed to equal dinoflagellates (where available) and diatoms where unavailable. The model was tuned by varying grazing intensity with scalar values (AMPDiatomGraze, AMPPicoGraze, AMPDinoGraze) of 16.1 for diatoms and 2.7 for picoplankton and 3.0 for photosynthetic flagellates.

	**Parameters**	**Values**	**Units**	**Reference**	**Title in SI Fig. 3**
Geochemistry	initial nitrate	16.1	µmol kg^{-1}	Rafter and Sigman [2]	Nitrate
Geochemistry	initial recycled N products	1.0	µmol kg^{-1}	Letscher et al. [3]	RecycledN
Geochemistry	initial dissolved iron	0.09	pmol L^{-1}	Kaupp et al. [4]	Fe
Diatom	0.1% light biomass N	0.003	µmol kg^{-1}	Taylor et al. [5]	DiatomN
Diatom	100% light biomass N	0.035	µmol kg^{-1}	Taylor et al. [5]	DiatomN
Diatom	Fe:C	12.30	µmol mol^{-1}	Twining et al. [6]	
Diatom	Fe:N	0.082	nmol µmol^{-1}	Calculated from Twining et al. [6] assuming Redfield C:N:P	
Diatom	Growth rate maximum	2.40	day^{-1}	CESM	
Diatom	kNitrate	1.0		CESM	
Diatom	kFe	0.07		CESM	
Diatom	Non-Graze mortality	0.12	fraction	CESM	
Diatom	Max Grazing	0.44		CESM	
Diatom	kGrazing	1.15		CESM	
Diatom	Detrital N export / loss	0.3	fraction	CESM	
Picoplankton	0.1% light biomass N	0.010	µmol kg^{-1}	Taylor et al. [5]	PicoN
Picoplankton	100% light biomass N	0.080	µmol kg^{-1}	Taylor et al. [5]	PicoN
Picoplankton	Fe:C	4.0	mol mol^{-1}	Calculated from Twining et al. [6] assuming Redfield C:N:P	
Picoplankton	Fe:N	0.03	nmol µmol^{-1}	Calculated from Twining et al. [6]	

				assuming Redfield C:N:P	
Picoplankton	Growth rate maximum	0.62	day^{-1}	CESM	
Picoplankton	kRecycledN	0.050		CESM	
Picoplankton	kFe	0.030		CESM	
Picoplankton	Non-Graze mortality	0.11	fraction	CESM	
Picoplankton	Max Grazing	0.49		CESM	
Picoplankton	kGrazing	1.15		CESM	
Picoplankton	Detrital N export / loss	0.1	fraction	CESM	
Flagellates	0.1% light biomass N	0.030	$\mu mol\ kg^{-1}$	Taylor et al. [5]	PicoN
Flagellates	100% light biomass N	0.120	$\mu mol\ kg^{-1}$	Taylor et al. [5]	PicoN
Flagellates	Fe:C	14.2	$mol\ mol^{-1}$	Twining et al. [6]	
Flagellates	Fe:N	0.094	$nmol\ \mu mol^{-1}$	Calculated from Twining et al. [6] assuming Redfield C:N:P	
Flagellates	Growth rate maximum	1.03	day^{-1}	CESM	
Flagellates	kRecycledN	1.0		CESM	
Flagellates	kFe	0.07		CESM	
Flagellates	Non-Graze mortality	0.12	fraction	CESM	
Flagellates	Max Grazing	0.44		CESM	
Flagellates	kGrazing	1.15		CESM	
Flagellates	Detrital N export / loss	0.1	fraction	CESM	

Supplementary Note | Berkeley Madonna model code. This includes differential equations, equations used to calculate flux rates, and initial conditions used to drive the model.

```
TOP MODEL

RESERVOIRS
  d/dt (nitrate) = - uptakeDiatomN
    INIT nitrate = initNO3
    LIMIT nitrate >= 0
  d/dt (DiatomN) = + uptakeDiatomN - lysisDiatomN
    INIT DiatomN = initDiatomN
    LIMIT DiatomN >= 0
  d/dt (Fe) = - uptakeFe + recycAllFe
    INIT Fe = initFe
    LIMIT Fe >= 0
  d/dt (RecycledN) = - uptakePicoN + recycDN + recycPicoN - uptakeFlagN
    INIT RecycledN = initRecN
    LIMIT RecycledN >= 0
  d/dt (PicoN) = + uptakePicoN - lysisPicoN
    INIT PicoN = initPicoN
    LIMIT PicoN >= 0
  d/dt (FeBiomass) = + uptakeFe - lysisAllFe
    INIT FeBiomass =
initDiatomN*QFeQNDiatom+initPicoN*QFeQNPro+(3*initDiatomN)*QFeQNDino
    LIMIT FeBiomass >= 0
  d/dt (RecycledFe) = - recycAllFe + recycFe
    INIT RecycledFe = 0
    LIMIT RecycledFe >= 0
  d/dt (detritalDiatomN) = + lysisDiatomN - recycDN - expDN
    INIT detritalDiatomN = 0
    LIMIT detritalDiatomN >= 0
  d/dt (detritalPicoN) = + lysisPicoN - recycPicoN - expPicoN + lysisDinoN
    INIT detritalPicoN = 0
    LIMIT detritalPicoN >= 0
  d/dt (detritalFe) = + lysisAllFe - recycFe - exportFe
    INIT detritalFe = 0
    LIMIT detritalFe >= 0
  d/dt (FlagN) = + uptakeFlagN - lysisDinoN
    INIT FlagN = initFlagN
    LIMIT FlagN >= 0

FLOWS
  uptakeDiatomN = DiatomN*uDiatom
  uptakePicoN = PicoN*uPico
```

uptakeFe = (uptakeDiatomN*QFeQNDiatom)+(uptakePicoN*QFeQNPro)+(uptakeFlagN*QFeQNDino)
recycAllFe = recycFe*ONFeRecycle
lysisDiatomN = (DiatomN*DeathRateDiatom)
recycDN = detritalDiatomN*(1-ExpNDiatomN)
expDN = detritalDiatomN*ExpNDiatomN
lysisPicoN = (PicoN*DeathRatePico)
recycPicoN = detritalPicoN*(1-ExpNProN)
expPicoN = detritalPicoN*ExpNProN
lysisAllFe = (lysisDiatomN*QFeQNDiatom)+(lysisPicoN*QFeQNPro)+(lysisDinoN*QFeQNDino)
recycFe = (1-ExportFe)*detritalFe
exportFe = detritalFe*ExportFe
uptakeFlagN = FlagN*uDino
lysisDinoN = DeathRateDino*FlagN

FUNCTIONS
uDiatom = IF uDiatomFe<uDiatomN THEN uDiatomFe ELSE uDiatomN
uDiatomN = uMaxDiatom*(nitrate/(nitrate+kNDiatom))
DeathRateDiatom = GrazingDiatom+MortalityDiatom
GrazingDiatom = (MaxGrazeDiatom*(DiatomN/(DiatomN+kGrazeDiatom)))*AMPDiatomGraze
uDiatomFe = uMaxDiatom*(Fe/(Fe+kFeDiatom))
uPico = (IF uPicoFe<uPicoN THEN uPicoFe ELSE uPicoN)*ONPro
uPicoN = uMaxPro*(RecycledN/(RecycledN+kNPro))
uPicoFe = uMaxPro*(Fe/(Fe+kFePro))
GrazingPico = (MaxGrazePro*(PicoN/(PicoN+kGrazePro)))*AMPProGraze
DeathRatePico = (GrazingPico+MortalityPro)
uDinoN = uMaxPro*(RecycledN/(RecycledN+kNPro))
uDino = (IF uDinoFe<uDinoN THEN uDinoFe ELSE uDinoN)
uDinoFe = uMaxFlag*(Fe/(Fe+kFeDiatom))
GrazingDino = (MaxGrazeDiatom*(FlagN/(FlagN+kGrazeDiatom)))*AMPDinoGraze
DeathRateDino = GrazingDino+MortalityDiatom

GLOBALS

DT=0.1
; Initial parameter variables

initNO3=16.1 ; umol N kg-1 (from [2])
initRecN=1 ; umol N kg-1 (from [3])
initFe=0.09 ; nmol Fe kg-1 (from [4])

initDiatomN=0.04 ; umol N kg-1 (values from [5])
initPicoN=0.02 ; umol N kg-1 (values from [5])

initDino=0.04 ; umol N kg-1 (values from [5])
initBiomassFe=0.04 ; nmol Fe kg-1 (calculated using Fe:N ratio below)

; Iron and nitrogen requirements

QFeQNDiatom=0.0815 ; nmol Fe /umol N. Based on Fe/C of 12.3 umol/mol (from [6])
QFeQNPro=0.0265 ; nmol Fe /umol N. Based on Fe/C of 4 umol/mol (calculated from [6])
QFeQNDino=0.0941 ; nmol Fe/umol N. Based on Fe/C of 14.2 umol/mol (from [6])

; Grazing values

MaxGrazeDiatom=0.219 ; CESM
MaxGrazePico=0.245 ; CESM

kGrazeDiatom=0.087 ; CESM
kGrazePico=0.087 ; CESM

MortalityDiatom=0.12 ;10% grazing loss/day plus 2% aggregation loss/day
MortalityPico=0.11 ;10% grazing loss/day plus 1% aggregation loss/day

kNDiatom=1 ; CESM
kNPico=0.05 ; CESM

kFeDiatom=0.07 ; CESM
kFePico=0.03 ; CESM

uMaxDiatom=2.5 ; adjusted to fit model biomass
uMaxPico= 0.62 ; adjusted to fit model biomass
uMaxDino= 1.01 ; adjusted to fit model biomass

; Export values

ExportFe=0 ; iron export from detrital pool off

ExpNDiatomN=0.3 ; equals a 30% loss from diatom detrital N pool
ExpNPicoN=0.1 ; equals a 10% loss from picoplankton and nanoplankton detrital N pool.

;On-Off and Amplitude variables

ONFeRecycle=1 ; when varied from 0 to 1, changes iron recycling from off to on

AMPPicoGraze=2.7 ; changes the amplitude of picoplankton grazing. Used to fit model biomass to observations.

AMPDiatomGraze=16.1 ; changes the amplitude of diatom grazing. Used to fit model biomass to observations.

AMPDinoGraze=3 ; changes the amplitude of nanoplankton grazing. Used to fit model biomass to observations.

Supplementary References:

1. Letscher, R. T. & Moore, J. K. Preferential remineralization of dissolved organic phosphorus and non-Redfield DOM dynamics in the global ocean: Impacts on marine productivity, nitrogen fixation, and carbon export. *Glob. Biogeochem. Cycles* **29,** 325–340 (2015).
2. Rafter, P. A. & Sigman, D. M. Spatial distribution and temporal variation of nitrate nitrogen and oxygen isotopes in the upper equatorial Pacific Ocean. *Limnol. Oceanogr.* **61,** 14–31 (2016).
3. Letscher, R. T., Hansell, D. A., Carlson, C. A., Lumpkin, R. & Knapp, A. N. Dissolved organic nitrogen in the global surface ocean: Distribution and fate. *Glob. Biogeochem. Cycles* **27,** 141–153 (2013).
4. Kaupp, L. J., Measures, C. I., Selph, K. E. & Mackenzie, F. T. The distribution of dissolved Fe and Al in the upper waters of the Eastern Equatorial Pacific. *Deep-Sea Res. Part II-Top. Stud. Oceanogr.* **58,** 296–310 (2011).
5. Taylor, A. G., Landry, M. R., Selph, K. E. & Yang, E. J. Biomass, size structure and depth distributions of the microbial community in the eastern equatorial Pacific. *Deep-Sea Res. Part II-Top. Stud. Oceanogr.* **58,** 342–357 (2011).
6. Twining, B. S. *et al.* Metal quotas of plankton in the equatorial Pacific Ocean. *Deep-Sea Res. Part II-Top. Stud. Oceanogr.* **58,** 325–341 (2011).
